# Evaluation of the implementation of the Montreal at home/chez soi project

**DOI:** 10.1186/s12913-014-0557-6

**Published:** 2014-11-28

**Authors:** Marie-Josée Fleury, Guy Grenier, Catherine Vallée

**Affiliations:** Department of Psychiatry, McGill University, Douglas Mental Health University Institute Research Centre Research Centre, Montreal Addiction Rehabilitation Centre - University Institute, 6875 LaSalle Blvd., Montreal, Quebec H4H 1R3 Canada; Douglas Hospital Research Centre, Montreal, Quebec H4H 1R3 Canada; Rehabilitation Department, Laval University, Quebec, Quebec GIV 0A6 Canada

**Keywords:** Implementation process, Hindering and enabling factors of innovation, Mental health, Homelessness, Supported housing, At home/chez soi project, Housing first program, Evidence based practices

## Abstract

**Background:**

Homelessness and mental disorders constitute a major problem in Canada. The purpose of the *At Home/Chez Soi* pilot project was to house and provide supports to marginalised groups. Policymakers are in a better position to nurture new, complex interventions if they know which key factors hinder or enable their implementation. This paper evaluates the implementation process for the Montreal site of this project.

**Methods:**

We collected data from 62 individuals, through individual interviews, focus groups, questionnaires, observations and documentation. The implementation process was analysed using a conceptual framework with five constructs: Intervention Characteristics (IC), Context of Implementation (CI), Implementation Process (IP), Organizational Characteristics (OC) and Strategies of Implementation (SI).

**Results:**

The most serious obstacle to the project came from the CI construct, i.e., lack of support from provincial authorities and key local resources in the homelessness field. The second was within the OC construct. The chief hindrances were numerous structures, divergent values among stakeholders, frequent turnover of personnel and team leaders; lacking staff supervision and miscommunication. The third is related to IC: the complex, unyielding nature of the project undermined its chances of success. The greatest challenges from IP were the pressure to perform, along with stress caused by planning, deadlines and tension between teams. Conversely, SI construct conditions (e.g., effective governing structures, comprehensive training initiatives and toolkits) were generally very positive even with problems in power sharing and local leadership. For the four other constructs, the following proved useful: evidence of the project’s scope and quality, great needs of services consolidation, generous financing and status as a research pilot project, enthusiasm and commitment toward the project, substantially improved services, and overall user satisfaction.

**Conclusion:**

This study demonstrated the difficulty of implementing a complex project in the healthcare system. While the project faced many barriers, minimal conditions were also achieved. At the end of the study period, major tensions between organizations and teams were significantly reduced, supporting its full implementation. However, in late 2013, the project was unsustainable, calling into question the relevance of achieving a significant number of positive conditions in each area of the framework.

## Background

The literature shows that access to housing and support interventions are effective weapons against homelessness [1,2]. One evidence-based practice that is considered effective for people with severe mental disorders and chronic homelessness is the “Housing First” program [3]. Contrary to the residential continuum model, where independent living accommodations are offered only after completion of particular rehabilitative programs of activities, Housing First programs provide immediate access to subsidised housing based on user preferences and ensures appropriate clinical follow-up [4]. In the Housing First program, housing is not dependent on treatment, and users who continue to abuse substances do not lose their lodging [1,5]. Introduced in New York in 1992 with Pathway to Housing [6], this program has since been successfully tried in various settings in the United States and other countries [5-8]. Nine randomised controlled trials have acknowledged the Housing First program as an evidence-based practice [3].

In 2008, the Canadian federal government allocated Can$110 million to the Mental Health Commission of Canada (MHCC) for the implementation of a four-year research pilot project to replicate and adapt the Housing First program (2009–2013). The *At Home/Chez Soi* project [4] was then launched in five Canadian cities: Vancouver (British Columbia), Winnipeg (Manitoba), Toronto (Ontario), Moncton (New Brunswick) and Montreal (Quebec). It provided access to three essential services: 1) affordable and safe housing, using rental money as support for housing units, monetary subsidies for certain landlords or offering housing units owned by the project; 2) assertive community treatment (ACT; multidisciplinary team follow-up including a psychiatrist offering services several times a week; one service provider full-time equivalents (FTE) for ten users) for homeless people with severe mental disorders having high needs; 3) intensive case management (ICM; individual follow-up by a case manager at least one time a week; one service provides FTEs to 20 users) for homeless people with severe mental illness having moderate needs. The recovery paradigm, dominant in the mental health field [9], where all decisions and interventions focus on user needs and where the users are a close partner of services, was also at the heart of the project’s vision and practice. As well, each local site could include components suited to their specific needs and conduct sub-studies focusing on key local issues, as long as such activities did not interfere with the core of the Canadian project.

In Montreal, the *At Home/Chez Soi* project appeared on a dynamic political scene. In 2008, the government of Quebec had established a parliamentary commission on homelessness, and an Inter-ministerial Action Plan on Homelessness (2010–2013) published in December 2009 had recommended identifying best practices to fight homelessness. This plan acknowledged that the Housing First program could be a promising avenue for long-time homeless people with severe mental disorders [10]. The Montreal project also responded to the changing mental healthcare context. In 2005, the Quebec Mental Healthcare Action Plan (2005–2010) set targets for housing services supported by ACT and ICM teams, and promoted the Housing First program as an innovative solution for the homeless with severe mental disorders [11].

However, the Montreal *At Home/Chez Soi* project was also lunched in a context of strong long-standing debates between the Quebec and Canadian governments about their respective jurisdictions. Starting at the turn of the last decade, the federal government had sponsored extensive, non-recurring health initiatives throughout Canada, which was later transferred to provinces without additional funding, thus adding pressure to provincial budgets. The Quebec government especially disapproved of federal involvement in health and social services, which are areas of provincial responsibility within the Canadian context.

In Montreal, the *At Home/Chez Soi* project aimed at recruiting 500 participants, including 300 in test groups receiving housing and clinical support (100 in each of the ACT and the two ICM teams). Control groups comprising 100 individuals for each level of need were formed for research purposes exclusively (no service were provided). Additional pilot projects, no required at the national level, were included, for example, the offer of both social and private housing choices to users. Monetary subsidies for private landlords or social housings were thus provided.

As opposed to Pathway to Housing in New York, which was a single organization, the Montreal *At Home/Chez Soi* project was sponsored by three principal partners: a mental health university institute (MHUI), a health and social service centre (HSSC), and a community agency. The MHUI handled the housing team and provided leading research expertise primarily in the mental health field. The Montreal project managers, i.e., the local *At Home/Chez Soi* coordinator (representing the MHCC, was responsible for ensuring the successful project implementation as planned at the national level and in accordance with appropriate adjustments at the site level) and the principal site investigator, were from the MHUI. The HSSC oversaw the ACT team and one of the two ICM teams, and brought complementary research expertise mainly to the social service and homelessness areas. The community agency managed the second ICM team.

Since the project involved several organizations and teams (ACT, ICM, Housing, user recruitment team) three governance structures were set up to integrate the project: a steering committee, an operational integration committee and a peer users council. The mandate of the steering committee was to vet strategic decisions of the *At Home/Chez Soi* project in Montreal. Under the direction of the site coordinator and principal investigator, it comprised a representative from each of the organizations involved in the project at all levels. The operational integration committee comprised team leaders (housing, clinical, recruiters), the staff psychiatrist (from the ACT team), and representatives from the peer users council, along with the site coordinator, principal investigator and research coordinator. The mandate of the operational integration committee was to oversee the operations of project components and the execution of the teams’ mandates. The role of the peer users council was to represent users’ points of view on the various project governance committees and to organise activities for them. It was constituted of individuals with lived experience of mental disorders and homelessness.

The Montreal *At Home/Chez Soi* project lends itself to an interesting study as its implementation involved a complex set of actors, including federal and provincial governments, MHCC and local governance structures, public (MHUI, HSSC) and community organizations, health and social services, stakeholders from the mental health and homelessness fields (clinicians, managers, researchers and users) and teams with specific mandates.

Implementation marks the transition between the planning of a new strategy or project and its acceptance as a regular program among all stakeholders [12,13]. It involves specific activities to meet established requirements of the project [14]. Implementation is a social process [12,15] in that it involves contextual factors, and organizations and individuals that contribute to its success or failure by their attitudes and actions (or inaction) [8,16]. It is difficult to effect any substantive change in health and social services systems. As the literature shows, almost two-thirds of such attempts fail [12]. This is why policy makers need to understand factors that can mean the difference between success and failure of new projects or services. Several conceptual models now exist that describe these factors [12,13,16-19]. Few studies however have looked at complex implementation processes related to the Housing First program [20,21].

This paper proposes to do just that based on an examination of the first implementation phase of the *At Home/Chez Soi* pilot project in Montreal, Canada (2009–2010). Basing ourselves on a conceptual framework, we will identify and comment on the foremost aspects that created roadblocks during the implementation of the Montreal *At Home/Chez Soi* project in order to achieve a clearer understanding of the dynamics of this process.

## Methods

### Setting

Montreal (Quebec) is the Canada’s second largest urban centre. According to the 2006 Census, it was home to 1.9 million people or 25% of Quebec’s total population. In 2006, 32.3% of households were below the low-revenue threshold, and 9.5% of the population received social welfare [22]. An estimated 30,000 individuals were homeless for at least part of 2005 [22,23]. There was a long tradition of cooperation and partnership on the issue of homelessness in Montreal before the arrival of Montreal *At Home/Chez Soi* project between the city, the health authority of Montreal, the HSSC and community organizations.

### Data collection

**Figure 1:**
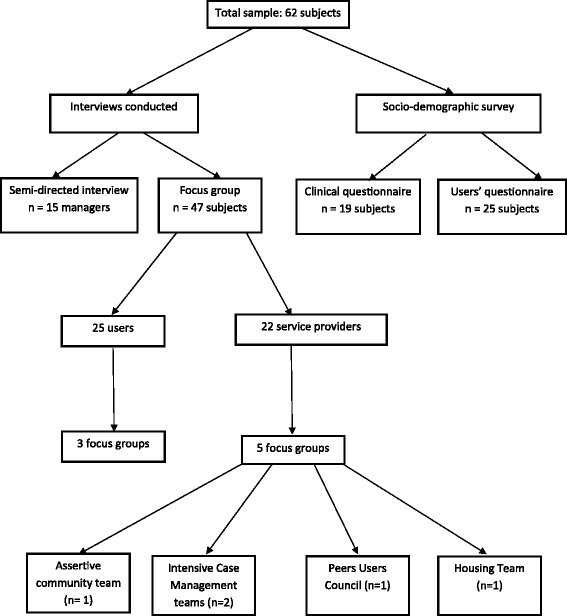
**Interviews flowchart.**

This research was a mixed methods study, using both qualitative and quantitative methods. The opinions of 62 various stakeholders were sought (service providers, decision makers, users, peer support workers and researchers) between October 2009 and December 2010. With the exception of the users, these included the main stakeholders involved in the Montreal project. They were chosen in view to reflect a diversity of opinion. Users were selected from each of the clinical teams by the team leaders, according to their availability and their varying degrees of commitment to the project. Figure 1 shows the flowchart of the study stakeholders and data collection used for each type of stakeholders. The 62 individuals surveyed, included 37 professionals and 25 users (from ACT and both ICM teams). The professionals were as follows: a) 15 managers, team leaders, psychiatrists or researchers in charge of the project; b) 19 service providers; and c) 3 representatives from the peer users council. The following methods were used: semi-directed interviews, focus groups, observations of meetings of the governance structure, and minutes of meetings of governing committees and questionnaires.

Qualitative investigations were used primarily to understand the implementation process [24]. The interviews and focus groups (except for users) covered the following dimensions: 1) implementation context of the *At Home/Chez Soi* project (e.g., team development, recruitment process); 2) role and operation of project teams and governing structures, including values and practices; 3) relationships across teams within the *At Home/Chez Soi* project and between the project and the local mental health and homelessness networks; 4) perceived impact of the project on users and homelessness; and 5) issues and challenges for the project. Users described their experience and appreciation of 1) their integration within the *At Home/Chez Soi* project including housing; 2) their clinical treatment (by key service providers, other professionals and external resources); and 3) the project in general (e.g., most useful aspects, needed improvements). Individual interviews took about 45 minutes; focus groups about two hours. Interviews and groups were recorded, transcribed and rendered anonymous, each participants being identified by a number.

Participants’ observations were realised throughout the entire period of the study within the project’s governing committees by the authors of this manuscript. The purpose was to observe interpersonal relationships between stakeholders as well as the level of leadership assumed by each of them. The minutes of the project’s governing committees completed the information of observations (e.g., subjects covered, actions taken, problem resolutions). The research also drew on correspondence related to the project. However, researchers did not observed interventions of clinical teams.

Quantitative data were used secondarily to complete qualitative data and for measuring intervention outcomes [24]. Three questionnaires were administrated (all quantitative data in the Results section are from those). First, all respondents received a questionnaire on socio-demographic data (e.g., education, time involved in the *At Home/Chez Soi* project). For clinical teams, we added the following items: training received during the period under study (number of days), work satisfaction (e.g., workload, work climate), and perceived impact of service providers’ intervention on users. This second questionnaire required categorical or continuous responses (yes/no, number or percentage), with some five- or ten-point Likert-scale questions (e.g., from very unsatisfactory to highly satisfactory). Lastly, in a third questionnaire, the users’ time spent with the project, users’ time in homelessness, and the reason why they lived on the streets was asked in addition to socio-demographic data.

The research response rate was 100%, i.e., all asked participants agreed to be part of our study. All participants also signed a consent form. The study protocol (MP-IUSMD-09-023) was approved by the Douglas Hospital Research Ethic Board Committee, the *Centre Hospitalier de l’Université de Montréal (CHUM)* Ethic Board Committee and the Jeanne-Mance Health and Social Service Centre (HSSC) Ethic Board Committee.

### Analyses

**Figure 2:**
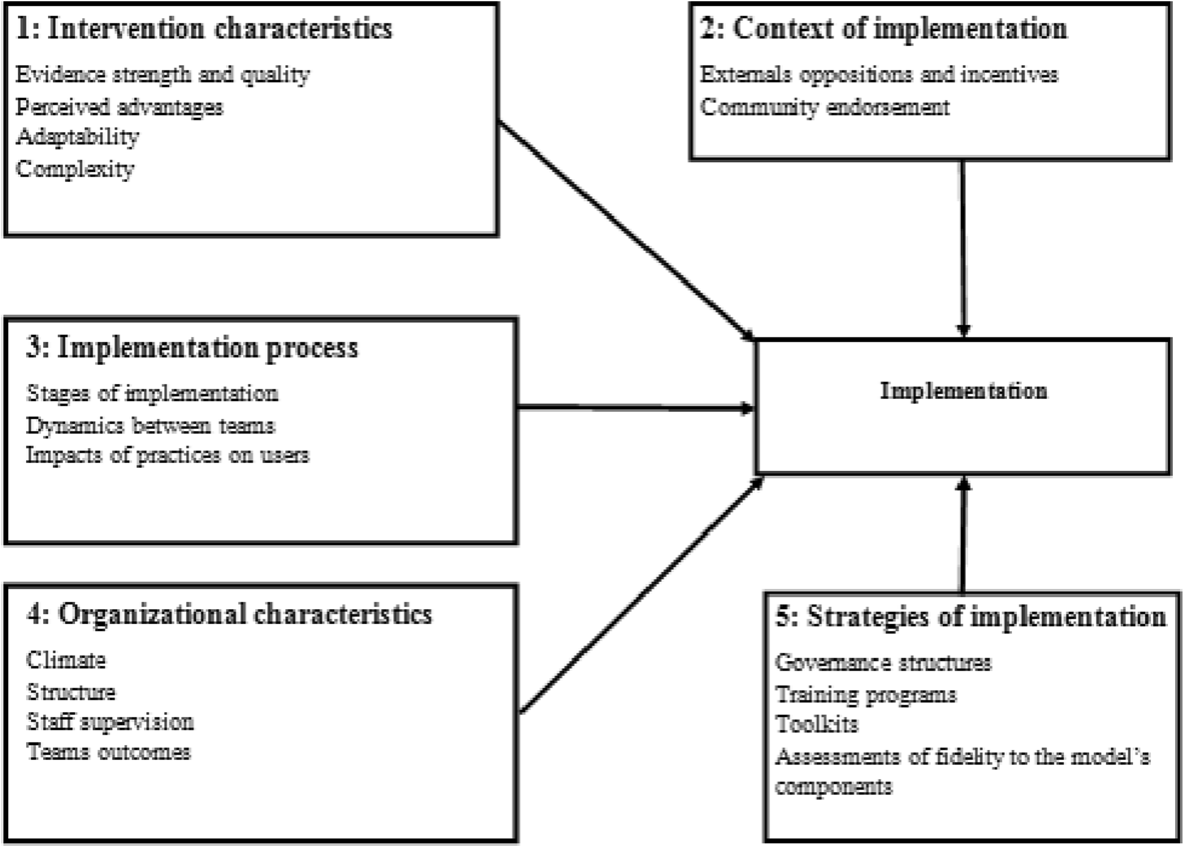
**Conceptual Framework.**

The qualitative data analysis used a thematic analysis method [24]. The initial coding structure was based on the general interview topics identified above, but allowed the inclusion of emerging issues such as sustainability, intergovernmental relations and other contentious aspects. SPSS software was used to compile quantitative data and produced descriptive analyses on questionnaire items according to type of participants, specifically providers or users. Univariate statistics comprised frequency distributions for categorical variables and mean values along with standard deviations for continuous variables. Information was triangulated across stakeholders, and types of data collection, including qualitative and quantitative methods. Results were drafted in a research report, validated by the main stakeholders, and subsequently submitted to the National Research Team [25] on which this article is based. The analysis was also guided by a conceptual framework based on previous models [12,13,16,17,19] and on the implementation literature [6,8,14], and elaborated on by the authors after consensus. Factors associated with implementation were grouped in five key areas, detailed in Figure 2.

## Results

The socio-demographic description of the 37 professionals is provided in Table 1, and those of the 25 users in Table 2. The results section that follows is based on the analysis of the strengths and weaknesses of the Montreal *At Home/Chez Soi* implementation process according to the five key areas of the conceptual framework: intervention characteristics, context of implementation, implementation process, organizational characteristics and strategies of implementation. Examples of interview quotes for each of those key areas are presented in Table 3.

**Table 1:** **Socio-demographic description of professionals**

**Variables**	The 4 teams* (n = 19)	**Managers/Team leaders/Researchers (n = 15)**	**Peer Council Members (n = 3)**	**Total (n = 37)**
**Average age (s.d.)**	38.93 (9.99)	46.87 (11.17)	36.64 (4.72)	42.33 (10.85)
**Gender (%)**				
Women	16 (84.2)	10 (66.7)	1 (33.3)	27 (73.0)
Men	3 (15.8)	5 (33.3)	2 (66.7)	10 (27.0)
**Average number of years since last diploma obtained (s.d.)**	5.80 (4.39)	14.07 (9.72)	12.50 (10.60)	10.09 (8.48)
**Years of acquired experience (s.d.)**				
Public health system	3.34 (4.57)	14.73 (11.79)	2.0 (2.0)	8.39 (10.23)
Community sector	8.50 (6.03)	8.06 (9.4)	2.0 (3.46)	8.20 (7.44)
Homelessness	4.67 (3.68)	8.40 (5.97)	4.11 (5.16)	6.31 (5.19)
Mental Health	9.01 (6.41)	14.13 (11.25)	2.33 (1.52)	10.74 (9.30)
Current workplace	0.85 (0.39)	22.0 (5.7)	0.75 (0.35)	3.12 (5.16)

*The 4 teams:community-organization- ICM, HSSC-ICM, HSSC-ACT, Housing team.

**Table 2:** **Socio-demographic data of users (N = 25)**

**Variables**	**CO-ICM (n = 10)**	**HSSC-ICM (n = 6)**	**HSSC-ACT (n = 9)**	**Total (n = 25)**
**Average age (s.d.)**	49.10 (6.00)	43.83 (12.84)	46.11 (10.30)	46.76 (9.37)
**Gender (%)**				
Women	3 (30)	3 (50)	2 (22.2)	8 (32)
Men	7 (70)	3 (50)	7 (77.8)	17 (68)
**Race (%)**				
White or Caucasian	8 (80)	4 (66.7)	9 (100)	21 (84)
Other	2 (20)	2 (33.3)	-	4 (16)
**Mother tongue (%)**				
English	3 (30)	4 (66.7)	2 (22.2)	9 (36)
French	6 (60)	2 (33.3)	7 (77.8)	15 (60)
Other	1 (10)	-	-	1 (4)
**Education level (%)**				
Primary school	3 (30)	2 (33.3)	2 (22.2)	7 (28)
High school	5 (50)	1 (16.7)	5 (55.6)	11(44)
College	2 (20)	1 (16.7)	-	3 (12)
University	-	2 (33.3)	2 (22.2)	4 (16)
**Average time spent with the project in months (s.d.)**	7 (3.39)	7 (2.19)	4.67 (2.39)	6.13 (2.89)
**Average time spent on the street in years (s.d.)**	6.28 (6.51)	5.50 (8.31)	9.73 (9.51)	7.41 (7.97)
**Main reasons why living on the street (%)**				
Poverty	5 (50)	2 (33.3)	-	9 (50)
Cost of housing	7 (70)	3 (50)	2 (22.2)	12 (66.7)
Physical health problems	3 (30)	1 (16.7)	1 (11.1)	5 (27.8)
Mental health issues	7 (70)	3 (50)	-	10 (55.6)
Substance-abuse issues (alcohol, drugs, gambling)	3 (30)	3 (50)	1 (11.1)	7 (38.9)
Other issues*	2 (20)	4 (66.7)	-	6 (33.3)

*Job loss, marital problems, legal problems.

**Table 3:** **Example of quotes**

**Intervention Characteristics**	
*Adaptability*:	“I am frustrated a bit by certain types of information, or certain ways of seeing things. I did not have a chance to explain how we, especially in Quebec in our specific culture, see things. I learned this later in life and when you react later, it is often not seen as it should have been seen.” (12-Manager)
*Adaptability:*	“Well, we came with a housing first approach, which really disrupted things […]. This is something different, so it always requires efforts on the part of those involved in spite of the information and everything. For some, it’s easy, and for others it’s more difficult. I think it is not over. One cannot acquire the skills and abilities in one year… there is still work to be done.” (31-Manager)
*Complexity*:	“For owners who were not aware, because they never had had previous experience, we took the time to explain our agreement to them, in what we were getting ourselves into, and told them that it was the person that we would introduce them to would be signing the lease, and that that person would be a tenant like everyone else, committed to his or her responsibilities and his or her rights on both sides, and that we were a team there to support them in this project, that we would be real partners. Yes, it is a bit long, because we really have to make this long-winded speech; we really have to fully inform the owners so that they knew what type of project they were embarking on.” (27-Housing Team)
*Conplexity*	“I think that this is a complex, large project […]. And I must say that I am still learning about this project. I still feel like I am learning about what is happening, what the issues are…” (32- Manager)
**Context of Implementation**	
*Opposition to Housing First*:	“In the community, in other organizations, we heard people speaking against it. It was a bit of a wave against the Housing First Project. It will be like taken over. They (the users) will be used. After that, they will be dropped, etc. There was a lot of prejudice in that regard.” (28- ICM Community Agency Team)
*Shock of culture*:	“It is the basis of the *Housing First* approach, the subsidies for rent in the private sector that they found morally wrong […]. It is their way of seeing the world, that many of us do not share…” (01-Manager)
*Incentives:*	“There were many meetings, and basically it was to generate some interest in those in the grassroots so that this project could come to Montreal […] to try to see how we could deal with the problems with intergovernmental affairs, the resistance of the Government of Quebec, and thus the Health and social services ministry and the Health regional agency regarding this project. Essentially, to develop an interest by the *grassroots*…” (04-Manager)
**Implementation Process**	
*Conflicts between the Housing Team and the Clinical Teams:*	“There is indeed an issue about the mandate, the housing team, their clients… they are the owners. We, our clients, they are participants who sometimes have a relationship with the owner that is not always satisfactory. When we speak, we do not have the same objective.” (11-Manager)
*Shock of cultures*:	“With the clinical team, it was more focused on the participant and the participant’s problems, and history. […] We, by definition the Housing Team, are more associated with the “Community”. We are not in the life of the participant. We deal with the owner, the territory, the resources, etc.” (27-Housing Team)
*Lack of Qualification of the Teams:*	*“*As nurses, this is also new. We do a lot of legwork, observation, evaluation, whereas we work far more now with the social worker, paperwork, the local job centre… all that for us is major. It’s new. We do not see that at school.” (49- HSSC ACT Team)
*Lack of Qualification of the Teams*:	“What is an issue between ICM and Housing, as with ACT and Housing, is that the Housing Team was not made up of people trained in mental health.” (30- HSSC ICM Team)
*Staff Turnovers:*	“We got to know them at the time, and then we had new stakeholders, and then… I mean, we had to start everything all over. Then… No matter how much they talk among themselves, but I mean, you know, it’s…” (User HSSC ACT Team)
*Loneliness:*	“So I will say like she did: “You say…” I will come back to that, the loneliness and then … The loneliness, and that’s it… At the start, for the first four months, it was hell.” (User ICM Community Agency Team)
**Organizational Characteristics**	
*Climate*:	“We are far smaller and more flexible machines. And we have always been flexible, we can easy turn around and I wonder whether we are more open […]. So, if there is a problem I think it is easier to talk about it and find a solution.” (05-Manager)
*Difficulty to integrate activities for big organizations:*	“You know, the HSCC is a strange machine. I can’t believe we have this in Quebec, but it’s a strange machine. It’s complex, you have very little autonomy, everything is regulated, even the furniture.” (03-Manager)
*Culture:*	“In fact, these problems, these are the problems with the entire project, i.e., that it is an ad-hoc alliance between several partners with philosophies from the outset that are not necessarily that similar, which have been brought together by the project and are starting off from very different traditions, which do not have a long history of working together, so we still talking about institutions that are not accustomed to working together, with each having its own particular culture.” (04-Manager).
*Positive Outcomes*:	“What we are in the process of creating that will remain is all the learning in terms of daily living activities and domestic life. We are teaching them ways of living in a healthier apartment setting. I think that will remain with them.” (44-HSSS ICM Team)
**Strategies of Implementation**	
*Loss of Meaning and Lack of Discretion:*	“I know that, in the first planning report that was produced as part of this study, there was an issue that there was not enough discretionary powers given to the local level during the planning and development phase. I did not think that there would be more latitude. And that we would still have to refer to the national level for fundamental questions. It is more the national level that provides direction.” (05-Manager)
*About the Steering Committee:*	“I do not personally believe that is vested managerial power. Because the real management issues are a matter for the national level. I believe this, because after all it is a multi-site party, so we are just one site among others, yes…” (07-Manager)
*About the Peer Users Council:*	“We did not really participate in the planning. It was a housing program first and foremost, and then the Housing Team came at the same time as us, so, to me, it was like you starting a car, and you did not have your two main wheels. Honestly, I really felt like that.” (18- Peer User Council)
*Expectation from the Peers Users Council*:	“The Peer Users Council was supposed to be really involved at the clinical level. That was at least what I thought, and it is not that at all […] Absolutely no one knows it. It’s sad […] and perhaps if we think of involving the participants, we are miles from there.” (47-S HSSC ACT Team)

1. *** Intervention characteristics*** – *evidence strength and quality, relative advantage, adaptability and complexity*

Since the *At Home/Chez Soi* project was an evidence-based practice, the project was strongly endorsed by the Montreal stakeholders, particularly the public organizations (MHUI, HSSC) and mental health network. Stakeholders perceived the project as a strong quality-based intervention, which could help reduce homelessness in the City of Montreal, especially for individuals dealing with severe mental disorders and chronic homelessness, who are more difficult to reach, and for whom few services are available. Moreover, the National Research Team, which spearheaded the project, as well as the local coordinator and principal investigator of the Montreal site, all three from the mental health field, brought to the project considerable expertise in the field of psychiatry. However, homelessness was a problem generally addressed by health and social service providers and community organizations, which might have made it more difficult to establish the legitimacy of the project.

In addition, as the *At Home/Chez Soi* project was a multiple-site research initiative sponsored at the national level (MHCC), it favoured standardised practices derived from evidence-based data, which had promoted an approach mostly viewed by stakeholders as very top-down. Appropriation of the project was thus more difficult at the site grassroots level, according to the majority of managers, since they felt that adaptations or local ways of implementing were negated. The MHCC sought high fidelity to the initial program and its many components (e.g., ACT, ICM, housing), including research, from all five settings. The fact, as well, that the project included several organizations contributed to its perceived complexity, hindering its implementation. Greater effort was therefore put on stakeholders to coordinate the project to answer the comprehensive needs of the users.

(2) *** Context of implementation*** – *external policies and incentives and community endorsement*

The Quebec government or its representatives took no official part in the Montreal *At Home/Chez Soi* project, which they felt encroached upon their constitutional jurisdiction, thus seriously hindering its sustainability. Moreover, the project was launched quickly with little consultation with the provinces, which did not help endorsement by the Quebec government. Community organizations, especially those active in the fight against homelessness or offering housing services, were also reluctant about the project, because it favoured private housing over social housing as the prime choice for users. Social housing has been historically the orientation approved in the homelessness field, which clashed with the rent-supplement orientation of HF.

Furthermore, the *At Home/Chez Soi* project had a bio-psycho-social approach, both in terms of combating homelessness and promoting mental health. This vision did produce a culture shock and pose challenges since stakeholders involved in each field of homelessness and mental health had their own history, values and ways of doing things. Mental health is grounded in the field of psychiatry and under the governance of the health branch of the Health and Social Services ministry, while the homelessness sector is grounded in social service and community organizations. Many stakeholders coming from the field of homelessness felt that the expertise and skills that they had acquired and developed over the years were summarily dismissed by this pilot project involving a consortium of providers who came mostly from the field of psychiatry. Nonetheless, given the *At Home/Chez Soi* project was a pilot project, financed by the federal government, and bringing major funds to the field ultimately generated support for it with the increasing needs and lack of resources to deal with homelessness.

(3) *** Implementation process*** – *stages of implementation and related dynamics and impacts*

The implementation process of the Montreal *At Home/Chez Soi* project can be divided into three distinct periods [26]. The first period (October 2009 to March 2010) involved recruitment of team staff and project users, followed by a two-month hiatus to give the MHUI time to draft an agreement with landlords allowing users to rent accommodations. During this period, clinical teams and recruiters for the project developed tools, approaches and strategies. The innovative character of the project posed a serious challenge for everyone. At this stage, research considerations were front and centre and set the pace for the project (recruitment and housing targets, and level of treatment activity). According to the great majority of managers and teams leaders, teams were dynamic, committed to the project’s success, and willing to provide valuable services to the homeless often disregarded by the healthcare system.

During the second period (April to August 2010), pressures to meet research deadlines were paramount, i.e., maintaining the pace of recruitment of users, rapidly finding housing for them, and providing the right intensity of service while recognising the long travel time for user visits by the staff. This situation contributed to team exhaustion and a crisis management mode. The social housing option, which was to be part of the study, had to be cancelled, resulting both of the difficulty in finding such accommodations for individuals with substance abuse issues, the virtual boycott of the project by organizations having these resources, and preferences of users for private housing. More demands were thus placed on the private sector, which made it increasingly difficult to find affordable housing.

The third key period (October to December 2010) saw further tensions between the housing and clinical teams, related to their respective responsibilities and mandates, and inadequate coordination between the teams. Under the *At Home/Chez Soi* project structure, the Housing team felt pressured to rapidly find apartments for newly recruited users. There was, however, a lack of coordination with the clinical teams whose job it was to assist these new users while providing the required intensity of service for those who were already settled. In addition, since the Housing team did not want to lose its stock of apartments, it was severely criticised by the clinical teams for defending the interests of landlords over those of users.

During an operational integration committee meeting, the Housing team maintained that when a user refused three or four apartments, he became a no-priority case. For the clinical teams, conversely, it was normal that a user visit several apartments before selecting one. Moreover, if a user expressed his will to move due to a dispute with his landlord or his neighbours, the clinical teams could be in favour of that, while the Housing team invoked the obligation of the user to respect bail. Other difficult challenges that teams had to deal with during this period included non-payment of rent or abandonment of housing accommodations, long delays in finding housing, having to develop intervention plans for users with complex profiles and largely unknown histories, repeatedly missed or cancelled appointments, refusal of treatment and having to serve a vast area. Nonetheless, near the end of this period, considerable efforts and gains led to the roll-out of strategies and conditions more likely to result in the successful implementation of the project. At the beginning of the winter of 2011, the Montreal site, with the agreement of the National Research Team, decided to reduce the high mental health needs cohort to 160 (before: 200), i.e., 80 individuals in each of the experimental and control groups.

(4) *** Organizational characteristics*** – *climate, structure, staff and teams outcomes*

Each of the three organizations that sponsored the project had its own culture, driven by their respective team function. Compared with the HSSC and the MHUI, the community agency had few staff and resources, and a flat hierarchical structure that encouraged administrative and procedural flexibility and ensured close supervision. The other two partners were large organizations, and thus found it difficult to integrate activities such as the hiring process and the introduction of new planning or follow-up tools within their structure. For exemple, according to HSCC managers, the intervention plan forms could not be adapted because such a change would have required approval by the archive service only after long and complex negotiations. As well, managers were not as accessible in the larger organizations, according to a few members of the clinical teams. This had considerable impact on team operations, especially the two HSSC clinical teams, which had to deal with high turnover and frequent understaffing.

The HSSC had to deal with the departure of both leaders of the ACT and ICM teams and later of the program manager, thus hindering ongoing supervision of the teams. Teams were to be completed progressively as new users joined the project. Under the MHUI’s direction, the Housing team was not fully staffed until March 2010 (n = 7 FTE). The community agency’s ICM team (five case managers FTE) was constituted from the very start of the *Montreal At Home/Chez Soi* project (fall 2009), and remained stable and fully staffed throughout the study period. The three participating organizations of the *At Home/Chez Soi* had also different levels of familiarity and background with Housing First and related community recovery concepts, resulting in an easier adoption of the latter approach by the community agency compared to the MHUI or the HSSC. The MHUI and the HSSC both entered uncharted territory, the former in trying to develop private housing, and the latter in providing ACT and ICM services for homeless people with severe mental disorders.

The questionnaire results from the service providers indicated mixed perceptions of the organizational features. Only a small majority of service providers were satisfied or highly satisfied with their inter-professional relations within their team (65%, n = 11), or their work climate (59%, n = 10). A minority reported being satisfied or highly satisfied about their team workload (29%, n = 5), since recruitment was intensive, and the demands for engaging and housing new participants were high. Conversely, 76% (n = 13) were satisfied or highly satisfied with their working conditions, 71% (n = 12) with their training, and 88% (n = 15) with the leadership of the *At Home/Chez Soi* project. Only 48% (n = 8) of service providers, however, expressed satisfaction with inter-professional relations with other project teams, and 56% (n = 9) with relations within the healthcare system. These numbers showed managers and teams leaders that most conditions within and across teams required significant improvement. The marked level of satisfaction among service providers with regard to the leadership of the *At Home/Chez Soi* project and, to a lesser extent, toward their working conditions, was nevertheless indicative of a strong commitment to the project.

In spite of these difficulties, 84% of the members of the clinical teams believed that their work was judged satisfactory or highly satisfactory by users. They felt that they had achieved a therapeutic alliance with 74% of the users they had served. Surveyed users also were generally satisfied with the help provided by the teams, although some noted that there was too much staff turnover, while a few reported having to wait too long for housing. These concerns were voiced in focus groups. Throughout the latter, the great majority of users nevertheless reported being concerned with key problems such as loneliness, social isolation, poverty and difficult integration within the community.

(5) *** Strategies of implementation*** – *governance structures, training programs, toolkits and assessments of fidelity to the program’s components*

### Governance

These different strategies were identified by stakeholders as key enabling factors in the project implementation process. In terms *of governance*, the National Research Team played a leading role in the Montreal site because of the need for standardization across sites, and because the majority of decisions having an impact on the research parameters had to be reported to them. This standardization led to a certain “loss of the project’s meaning” and to a certain disinterest among some local stakeholders, who were unsatisfied with their role as essentially project operators.

At the Montreal site, the coordinator exerted considerable control, acting as a buffer between the various interest groups within the project (e.g., clinical, organizational, research, users, and national/local). This power, however, was more persuasive than authoritative, since there was no hierarchical control over the organizations involved in the project. During this implementation phase, the local coordinator was also the head of the Housing team, which was the focus of much criticism and conflicts with the clinical team leaders. The neutrality of such a position was a key issue, and at the end of this study period, the steering committee recommended that the coordinator be appointed full time, and that a new head be nominated for the Housing team. The coordinator exercised leadership within a two-headed structure involving also the Montreal principal investigator.

A significant number of people thought the steering committee’s mandate was unclear. The in-between position of the steering committee relative to the National Research Team and the operational integration committee made it even more difficult to define its mandate, and thus the steering committee ended up playing more of a consultative role. Conversely, the majority of stakeholders considered that the operational integration committee was the most inclusive and successful committee given its ability to achieve results and resolve tensions. It considered information about what worked well and difficulties met along the way to reach consensual solutions. Concerning the peer users council, its members regularly attended the meetings of all the project’s committees and took real ownership of the issues, understood them fully and acted as effective advocates, according to the majority of managers. However, the peer users council did not follow through on proposed activities, remained little known among users and then failed to deliver up to expectations. According to the peers themselves, this state of affairs was the result of the council not having been involved closely enough in the project’s planning and thus not having had the necessary tools to conduct its activities.

### Training programs

During the period under study (essentially 2010), teams also benefitted from extensive training, webinars and coaching, as needed. Communities of practice emerged across sites and served to improve the teams’ functional capacity and practices. On average, team members had 10.4 days of mental-health-related education, 9.7 days of training in ACT or ICM techniques and 5.1 days of instruction about homelessness. According to most service providers, training fostered a sense of belonging to the project and among the newly constituted teams. Constant staff turnover among HSSC teams hindered learning activities, however, as did the need to meet urgent needs (e.g., user’s crisis) while trying to integrate new concepts.

### Toolkits

Various toolkits were developed to support the *At Home/Chez Soi* project. The Housing team introduced a list of vacant housing units, a description of each unit (e.g., number of rooms, brightness level), a quality evaluation form (e.g., safety, cleanliness), a spreadsheet on the percentage of rent to pay, a photo gallery of apartment dwellings and a geographical map showing their location. Meanwhile, the clinical teams, especially the community agency ICM team, developed a scale of readiness to change and other instruments such as crisis plans, records of user needs, life stories, neighbourhood maps including resources available for users (e.g., food banks, day care centres). The toolkits developed by HSSC teams were more formal given they had to follow the institution’s established standards of clinical practice (e.g., use of computer resources, intervention plans). All teams favoured motivational interviewing and a strength-based approach, but this was especially true of the community agency ICM team. In addition, new team recruits were always paired with another professional so as to adapt to the different aspects of the work. While all teams prioritised in-house training, especially role-playing, staff turnover at the HSSC undermined the appropriation of the concepts and approaches put forward by the *At Home/Chez Soi* project.

### Fidelity of the program’s components

During the third implementation phase in the fall of 2010, the National Research Team conducted an assessment of fidelity to the program’s components [3]. This evaluation created expectations and subsequent tensions between teams, although it had been meant to be followed up upon to improve team functioning. A consultant specialised in ACT and ICM was also hired in an effort to define more clearly the teams’ duties and improve coordination between them. Although the four team leaders met regularly at the operational integration committee, and had occasional conversations, the information, according to the majority of stakeholders, did not trickle down systematically to service providers. There was no formal mechanism or boundary spanner to bring the teams closer together. Nonetheless, at the end of the period under study, the majority of stakeholders agreed that there were positives changes with respect to the overall synergy between the project components (e.g., consolidation of HSSC teams, improved task distribution among teams and governing structures, clarification of the peer users council’s mandate; full-time employment of the site coordinator and a better neutrality of such function).

## Discussion

This study analysed the initial phase (October 2009 to December 2010) of the implementation process of the Montreal *At Home/Chez Soi* project, offering housing and community follow-up to homeless individuals with severe mental disorders. Using a conceptual framework including five key areas, the crucial aspects that created roadblocks during the project’s implementation were identified. The results confirmed the presence of positive and negative factors in each of the five key areas.

Concerning the *Intervention Characteristics*, the experience of the *Montreal At Home/Chez Soi* project showed that an evidence-based practice with obvious strengths and advantages does not automatically lead to success [16]. Implementation always involves negotiations with stakeholders. If the project is top-down, as was the case for *At Home/Chez Soi,* its supporters have to argue a strong case for gains to be made and needs to be fulfilled [27]. In this instance, the severe needs of the homeless and the lack of services were serious considerations in favour of the project’s implementation. The complexity of the *At Home/Chez Soi* project, however, constituted a significant obstacle to its implementation [16]. It involved the recruitment within a short range of 500 users, 300 of whom were to have access to housing of their choice and receive the services of an ACT or ICM team according to the severity of their mental disorders. Any delay could jeopardise the entire process. Moreover, the project could not be fragmented into more manageable parts and progressively implemented [16]. The literature tells us that simple innovations are more likely to be well received and successfully implemented [16,28]. Another major barrier to the implementation of the *At Home/Chez Soi* project was its operation as a multiple-site research pilot project [12,29] and its top-down approach conceived at the national level, which left relatively little leeway for adjustments at the local level. According to the literature, it is easier to start a new venture if local resources are brought to bear on the process [16,30].

It was within the *Context of Implementation*, however, that serious barriers to the implementation of the *Montreal At-Home/Chez Soi* project were most evident. It is strongly acknowledged that a positive relationship with government or mental health authorities facilitates the implementation process [27,31]. While the Montreal *At-Home/Chez Soi* project could rely on the MHCC as a firm champion, it met resistance from the provincial government, which hampered its success. Project promoters presented it as “the solution” to homelessness, and a better approach than social housing. This did not play well in Quebec where there is widespread support for social housing programs. There was a real clash of cultures among professionals. Stakeholders involved in the fight against homelessness felt that their expertise and long experience were being dismissed by the project promoters who worked primarily in the field of psychiatry [26]. In a previous study concerning the implementation of the Housing First program in an American suburban county, Felton [6] found that this program was also described as a new practice having unique expertise, and this again met resistance from local authorities. In Montreal, key community organizations that were active players in the homelessness field opposed the project, and much work has been invested in view to persuade them to not boycott the project [26]. This brought to the fore the importance of considering the views of external networks and sustaining healthy relationships with them to ensure the success of a new undertaking [12,32]. Advocates of innovations, like Housing First, and government authorities should plan and anticipate these tensions.

With regard to the *implementation process*, several authors have determined that it occurred in different stages and did not follow a linear trajectory. For instance, Greenhalgh and colleagues [16] identified three stages of implementation: knowledge-awareness, evaluation-choice and adoption-implementation. Fleury et al. [27] refer to these as “problem-setting”, “direction-setting”, and “structuring”. The *At Home/Chez Soi* project also followed three stages of implementation. The first was characterised by the firm belief that the project and the massive investments it entailed could achieve significant results in terms of user recovery. This could be seen as the “honeymoon and strong-support phase” of the project. Then came the “pressure-to-achieve and problem-solving phase” when the project’s implementation gathered momentum and expectations grew. The third phase of “crises and adaptation” evolved out of the collapse of the second phase, but brought accommodations leading to much optimism over the project’s implementation and achievements. Over the full implementation process, users were highly satisfied with the project but faced struggles such as loneliness, poverty, and difficult community integration.

Serious barriers to the implementation of the Montreal *At-Home/Chez Soi* project were also evident within the Organizational Characteristics. This pilot project was innovative in that it brought together three leading organizations and promoted collaboration with a large network. It can be described as a virtual integration program, i.e., a set of service providers having to coordinate their actions to offer diversified and ongoing services to a specific clientele [33-35]. These organizations nonetheless differed extensively in terms of structure, values and practices, which is why it was difficult to create an esprit de corps among various teams. This experiment shows—and the literature confirms [16]—that implementation is easier when organizations have some structural flexibility. According to Rosenheck [36], large organizations are often characterised by conflicting goals and inconsistent participation of key actors. It is also easier for organizations, such as the community agency involved in the Montreal *At-Home/Chez Soi* project (ICM), to offer their cooperation if their values align with those of the new initiative [31,37]. Moreover, the quality of the providers’ supervision had a significant positive effect on the implementation process [38]. Previous studies have found that frequent and abrupt turnover of supervisors or staff seriously hinders implementation [32,38,39]. The other side of the coin is that turnover can lead to the hiring of more willing and competent staff [32]. Finally, the experience of the Montreal *At-Home/Chez Soi* project confirmed the necessity of effective communication between services providers, teams and the network involved [40]. According to the literature, clear communication of mission and goals among the various providers and positive relationships between them promotes cooperation, which contributes to the ultimate success of an endeavour [12,39,41-43].

Regarding the *Strategies of Implementation*, the literature and the project’s history both attest to the positive impact of strong leadership [38]. According to Brunette et al. [31], successful projects tend to benefit from the active participation of mid-level managers. In the case of the Montreal *At-Home/Chez Soi* project, the operational integration committee exercised leadership at the operational level, but the absence of true strategic leadership able to promote local interests at the national level posed a serious barrier to the project’s implementation and resulted in disinterest among some local stakeholders. The experience of the Montreal *At-Home/Chez Soi* project also confirms the value of staff training [16,31] in the success of the implementation process. Training contributes to the propagation of knowledge, allows staff to become familiar with the tools needed for effective functioning, and, consequently, improves the confidence of employees in their ability to perform their job [12], and also fosters unity among team members [26]. To be effective, however, training and information dissemination need to be integrated with strategies encouraging the acquisition and maintenance of sound practices, coupled with coaching [14]. Many studies have emphasised this aspect [27,44] and have shown it to be at least as valuable as planning and other clinical or administrative procedures at the strategic and operational levels. The *At Home/Chez Soi* project involved considerable effort in that regard. Lastly, project evaluation such as fidelity assessment serves to take in account challenges in the implementation process and make necessary corrections. While there is no denying the importance of this step, it can still be a source of severe stress [12,39].

## Conclusions

This study of the Montreal *At-Home/Chez Soi* project demonstrated the difficulty of implementing a complex and new program in the social and healthcare system. While the project faced many barriers, minimal conditions were also achieved. At the end of the period under study, major tensions between organizations and teams were significantly reduced, which support its full implementation. However, at the end in 2013, although it had positive user impacts [45,46], the Montreal *At-Home/Chez Soi* project unfortunately was unsustainable, which calls into question the relevance of achieving a significant number of positive conditions in each area of the conceptual framework. In the specific case of the Montreal *At-Home/Chez Soi* project, most hindering factors stemmed from the context of implementation, followed by organizational characteristics, intervention characteristics, implementation process and strategies of implementation.

While there are limitation in generalizing our results to other studies on implementation, the Montreal *At-Home/Chez Soi* project thus served to emphasise the importance of identifying all the conditions that could hinder or enable a project and trying to fix most negative aspects before launching a project. It also showed that the success of a project depends largely on achieving the following conditions: support of the key actors within the social network, especially government authorities and long-term coalitions in the field, adaptation of the project at the site level, and compatible visions and approaches among project stakeholders. Other factors of successful project implementation are close supervision and support of staff at all hierarchical levels, human resources stability, collaboration among teams and with the social network (promotion of boundary spanners) and adequate training and effective deployment and integration of tools into practices. Others are related to the governance of the project and the various levels of authority, namely a clear definition of the mandate of each authority, and collegial distribution of power among stakeholders to let them play meaningful roles.

## Abbreviations

ACT: Assertive community treatment
FTE: Full-time equivalents
HSSC: Health and social services centre
ICM: Intensive case management
MHCC: Mental health commission of Canada
MHUI: Mental Health University Institute
